# Psychological Assessment via the Internet: A Reliability and Validity Study of Online (vs Paper-and-Pencil) Versions of the General Health Questionnaire-28 (GHQ-28) and the Symptoms Check-List-90-Revised (SCL-90-R)

**DOI:** 10.2196/jmir.9.1.e2

**Published:** 2007-01-31

**Authors:** Miguel A Vallejo, Carlos M Jordán, Marta I Díaz, María I Comeche, José Ortega

**Affiliations:** ^1^Facultad de PsicologíaUniversidad Nacional de Educación a Distancia (UNED)MadridSpain

**Keywords:** assessment, Internet, online

## Abstract

**Background:**

Internet psychology services are rapidly increasing and that implies online assessment. To guarantee the results of these new online evaluation procedures, it is necessary to have reliable and valid assessment tools.

**Objective:**

In this work we analyzed the online versions of two popular psychopathology screening questionnaires: the General Health Questionnaire-28 (GHQ-28) and the Symptoms Check-List-90-Revised (SCL-90-R).

**Methods:**

A total of 185 psychology students were recruited from two universities in Madrid, Spain. All of them had Internet access at home. A test-retest situation and factorial analysis were used to generate reliability and validity data. Both paper-and-pencil questionnaires (test) and their online versions (retest) were completed by 100 participants (median gap = 17 days).

**Results:**

Results suggest that both online questionnaires were fairly equivalent to their paper-and-pencil versions, with higher reliability values for the SCL-90-R. Factorial analysis tended to reproduce the structure shown in former investigations of both questionnaires, replicating the four-factor structure of the GHQ-28 but failing to do so with the nine-factor structure of the SCL-90-R. Instead, a large unrotated factor appeared.

**Conclusions:**

Further research should be carried out to confirm these data, but our work supports the online use of both assessment tools. The psychometric properties of the online version of GHQ-28 is similar to the paper-and-pencil and we can recommend its utilization in a Web environment. In contrast, SCL-90-R can only be recommended as a global index for psychological distress, using the Global Severity Index (GSI), not necessarily its subscales; and it should be considered that the online scores were lower than the ones with the paper-and-pencil version.

## Introduction

Nowadays, online assessment is becoming necessary as clinical psychology is considering the Internet as a medium through which therapy and counseling can be offered [1]. It has already been shown how easy it is to create a website containing tools to assess psychological problems or constructs [2]. Moreover, the advantages over the traditional way of gathering data, such as easy and immediate scoring and missing data handling, have been made evident [3]. At this point, the reliability and validity of online questionnaires have become current and relevant research topics.

So far, the question “Will the mode of administration affect the respondent’s score?” has barely been formulated, and research on this topic has been undertaken by only a few studies. Concepts related to social desirability [4], self-disclosure [5], or computer anxiety [6] are suggested as modulating variables that could modify the attitude toward computerized tests. Despite literature on these subjects, the research is still scarce and inconclusive and points to the need for further research to compare data from paper-and-pencil and online versions.

In that sense, a growing number of computerized or online questionnaires related to areas such as panic/agoraphobia [3], youth independence living [7], aggression and impulsivity [8], quality of life in diabetes [9], and a battery of 16 other health-related questionnaires [10] have already been studied. All but one of the computer/online versions (the Aggression Questionnaire by Buss and Perry [11]) were declared equivalent to their respective paper-and-pencil tests. Along with this, randomized studies on psychological distress tests have shown the same equivalence between the online and paper-and-pencil versions [12]. Nevertheless, in spite of the positive results supporting online assessment, the study of psychometric properties of online tests has frequent methodological problems (lack of random assignment or differing demographic characteristics to ensure sample equivalence), which make the adequate reliability or equivalence analysis difficult [13].

Taking the current state of the research into account, the present work aimed to obtain reliability and validity data for the online versions of two of the most frequently used psychopathology screening questionnaires in mental health: the General Health Questionnaire-28 (GHQ-28) [14] and the Symptoms Check-List-90-Revised (SCL-90-R) [15]. This paper is part of more extensive research aiming to develop a psychological treatment website following previous analysis of online clinical psychology websites in Spain [16]. Both questionnaires are used in a counseling website, the preliminary phase of the psychological treatment site. This choice was based on the wide research on and the historical use of these two questionnaires in psychopathology [17], as well as by their simple self-report structure, which makes it easy to incorporate them into a website.

A test-retest situation was chosen to obtain the reliability and validity data. Reliability was calculated as internal consistency, and test-retest correlation served as an equivalence index of the two test administration methods (paper-and-pencil and online). Inner structure exploration by factorial analysis was used to evaluate the construct validity of online versions. Although both questionnaires have a general score, they are divided into scales proposed as psychological disorders markers. The four scales of GHQ-28 (A: somatic symptoms, B: anxiety/insomnia, C: social dysfunction, and D: depression) have been found as a four-factor structure in previous studies [18-20]. For SCL-90-R, its nine scales (somatization, obsessive-compulsive symptoms, interpersonal sensitivity, depression, anxiety, hostility, phobic anxiety, paranoid ideation, psychoticism) were originally proposed as representing a nine-factor structure [21], but most of the research to date has failed to replicate this and has instead found either a primary global distress factor [22-26] or a four-, five-, or six-factor solution [26].

In short, with this work we try to contribute some of the needed empirical supporting data in order to ensure that online questionnaires have at least the psychometric characteristics attributed to their corresponding paper-and-pencil versions.

## Methods

### Sample

Participants were 185 psychology students recruited from two universities in Madrid, Spain. All of them had Internet access at home. This was a requirement to participate in the study in order to informally control how familiar participants were with the required technology. Although Internet familiarity is not a representative feature of the general population in Spain, this work is framed into a project in which the final point will be the development of a treatment website for mood disorders, so the sample resembles the target population in Internet familiarity.

### Instruments

#### GHQ-28

The General Health Questionnaire (GHQ) is used to detect psychiatric disorder in the general population and within community or non-psychiatric clinical settings such as primary care or general medical outpatients. In the GHQ-28 the respondent is asked to compare his recent psychological state with his usual state. It is therefore sensitive to short-term psychiatric disorders but not to long-standing attributes of the respondent. All items have a 4 point scoring system using Likert scoring (0-1-2-3). The GHQ-28 contains 28 items that, through factor analysis, have been divided into four subscales, as mentioned above. 

The Spanish-language version of the General Health Questionnaire by Lobo and Muñoz [28] was used. In the online version, one could scroll through the whole test. A pull-down menu in which the possible answers appeared followed the text of each item.

#### SCL-90-R

The Symptom Checklist-90-R (SCL-90-R) instrument has been designed to evaluate a broad range of psychological problems and symptoms of psychopathology. The instrument is also useful in measuring patient progress or treatment outcomes.

The SCL-90-R has 9 subscales, as mentioned above and in Table 4. The sum of all 9 subscales is the Global Severity Index (GSI), which can be used as a summary of the test, reflecting overall psychological distress.

We used the Spanish-language version of the Symptoms Check-List-90-Revised by González de Rivera et al [29]. The same online display method was used as for the GHQ-28.

### Procedure

A classic test-retest design was carried out: the paper-and-pencil version of the instrument was used for the test and the online version for the retest. After verbally agreeing to participate in the study, participants received a booklet containing instructions, sociodemographic questions, and both screening questionnaires in paper-and pencil-format. At the end of the instructions page there was a box with the address of the website containing the online questionnaires and the dates the site would be available. Identification of participants’ online questionnaires was achieved by a nickname chosen and written by each subject in the questionnaire booklet.

To ensure that participants completed the online tasks, email addresses were requested in order to provide reminder messages (22 participants refused). Individual messages were sent 14 days after the paper-and-pencil task. A second reminder was sent if the online questionnaires were not received within a week after the first message.

### Statistical Analysis

Statistical analysis was carried out with SPSS version 12.0. Reliability as internal consistency measured by Cronbach alpha was tested for both formats of the questionnaires and their subscales. Pearson correlation was used to prove the equivalence between paper-and-pencil forms and the online versions. A *t* test served to evaluate if there were statistically significant differences between the mean scores of the formats. We also applied η^2^ after a repeated measures ANOVA.  η2 is a measure of effect size in ANOVA: the degree of association between an effect (e.g., a main effect, an interaction, and a linear contrast) and the dependent variable. We used this statistic in trying to decide whether mean score differences have clinical relevance. Different benchmarks have been used to interpret η^2^, but as for the *P* < 0.05 rule in hypothesis testing, there is only a rough guide to be used when no literature is available to compare effect size values, and the best way to interpret it must consider what outcome is being studied [30]. As this “rough guide,” we will use η^2^ = .01 – .09 for a small effect, η^2^ = .10 – .24 for medium effects, and η^2^ ≥ .25 for large effects [31].

As stated earlier, construct validity was evaluated by means of principal components factorial analysis. Factorial structures similar to the ones shown in previous investigations following varimax rotation were expected, that is, four factors in GHQ-28 and nine in SCL-90-R. We also analyzed the unrotated solution and the sampling adequacy, using the Kaiser-Mayer-Olkin (KMO) test.

## Results

### Participant Demographics

From the initial sample of 185 participants, 104 completed both online questionnaires. This represents 56% of the total sample and 63% of those who received reminder messages. Although missing data was not possible online, four participants were rejected because of paper-and-pencil missing data, so 100 questionnaires were actually analyzed (Table 1). The majority of retests were received around the 14th day after the test (median = 17 days; min = 14, max = 38), and 90% had been received after 28 days.

**Table 1:** Demographic characteristics of the participants who completed all questionnaires, N = 100

**Demographic characteristic**	Number^*^
Gender	
Male	22
Female	78
Age (years), mean (SD)	27.4 (10.01)
Number of children, mean (SD)	0.28 (0.72)
Marital status	
Never married	83
Married	17
Education	
Technical education	3
High school	51
Short-term graduate	24
Long-term graduate	20
Undetermined	2
Socioeconomic status	
High	1
Middle-high	29
Middle	61
Middle-low	4
Low	3
Undetermined	2
Employment	
Full-time student	64
Worker/student	32
Unemployed/student	4

^*^Values are number unless otherwise noted.

### GHQ-28

Reliability results for the GHQ-28 are shown in Table 2. Cronbach alpha was .90 for the whole test in both the paper-and-pencil and online formats, and it ranged from .71 to .85 among the scales, with scale C (social dysfunction) showing lower values in both formats.

**Table 2:** GHQ-28 reliability results: Cronbach alpha, mean differences t test, Pearson test-retest correlation, and squared eta effect size

	**Paper-and-Pencil**		**Online**				
**GHQ-28**	**Mean (SD)**	α	**Mean (SD)**	α	***t* (*P* value)**	***r* (*P* value)**	**η^2^**
Scale A	4.77 (3.53)	.84	4.32 (3.48)	.84	−1.32 (.190)	.53 (< .001)	.017
Scale B	4.86 (3.80)	.83	4.19 (3.35)	.79	−2.45 (.016)	.72 (< .001)	.057
Scale C	6.81 (2.33)	.71	6.91 (2.21)	.79	.37 (.710)	.30 (.002)	.001
Scale D	1.27 (2.35)	.85	1.21 (2.51)	.84	−.30 (.769)	.65 (< .001)	.001
Total	17.71 (9.13)	.90	16.63 (9.00)	.90	−1.523 (.131)	.69 (< .001)	.023

Test-retest data showed significant correlations, ranging from .30 for scale C to .72 for scale B. Total score test-retest correlation was .69. We did a *t* test to see whether differences between scores from the two formats appeared. This occurred in scale B—paper-and-pencil scores were higher than online scores. We then used η^2^ to check how big this difference was if taken as an effect size: its value was small (.057), being in the same range as for those scales in which mean differences were not statistically significant (see Table 2).

The factorial analysis of GHQ-28 reproduces fairly well the presupposed four-factors solution in both the online and paper-and-pencil administrations. Table 3 represents item factorial loads among factors. Taking .30 or larger loads to assign each item to a factor, in both the online and paper-and-pencil analysis factor 1 includes all depression items (D), with the exception of online item D5, and factor 4 includes all social dysfunction (C) items, except paper-and-pencil item C2. Factor 2 grouped B (anxiety) items online and A (somatization) paper-and-pencil items. Factor 3 does the opposite, corresponding to A items online and B items in paper-and-pencil, except for B5. So, it could be said that each factor is close to its clinical interpretation. Nevertheless, a few items have bigger loads than expected in other factors. Scales A and B share large loads, a fact quite understandable given that somatization and anxiety appear together several times. Item D5 did not load at all in factor 1 in the online version, but did in factors 2 and 3. This could be explained by the meaning of the word “nerves” (included in the text of this item) identifying closer to anxiety than to depression. Item D5’s large load on paper-and-pencil factor 2 supports this interpretation. Lastly, paper-and-pencil scale C has smaller loads than expected in factor 4 in three of its seven items. We will interpret this alongside scale C’s test-retest correlation later.

The predominantly positive values in the original correlation matrixes suggest paying attention to a general unrotated factor that could explain some of the item sharing among scales. This general factor explained 28.44% (paper-and-pencil) and 29.48% (online) of the variance, and 27 and 26 (paper-and-pencil and online, respectively) out of 28 items had loads of .30 or greater.

**Table 3:** GHQ-28 item factorial loads (varimax rotation): A: somatization, B: anxiety; C: social dysfunction; D: depression

	Paper-and-Pencil (53.98%)^*^				**Online (55.85%)**			
**Item**	**1** **(16.18)^†^**	**2** ** (14.54)**	**3** **(13.59)**	**4** **(09.68)**	**1** **(16.07)**	**2** **(14.33)**	**3** **(13.06)**	**4** **(12.42)**
A1	.069	**.607^‡^**	.007	**.313**	.000	**.327**	**.416**	**.449**
A2	-.056	**.687**	**.328**	.282	.025	**.502**	**.486**	.254
A3	.121	**.759**	**.361**	.119	.180	**.591**	**.460**	.251
A4	.166	**.771**	-.066	.191	.290	.237	**.613**	.215
A5	.040	**.574**	.294	-.143	.097	.127	**.657**	.029
A6	.128	**.669**	.197	-.232	.045	.076	**.744**	.033
A7	.181	**.603**	.068	-.066	.236	**.326**	**.471**	.036
B1	.056	**.332**	**.575**	.153	**.303**	**.653**	-.071	-.036
B2	.029	**.317**	**.360**	.270	-.001	**.391**	.126	-.050
B3	.253	.218	**.796**	.017	-.040	**.722**	.207	.110
B4	.212	.067	**.815**	.090	.130	**.703**	.248	.240
B5	**.390**	**.363**	.226	-.141	.000	**.322**	**.547**	-.004
B6	.210	.078	**.775**	.113	.343	**.490**	**.315**	.179
B7	.182	.176	**.716**	.032	.111	**.719**	.271	.120
C1	-.055	-.084	-.224	**.501**	.213.	-**.337**	.195	**.578**
C2	**.456**	.291	.206	.213	**.422**	.074	-.086	**.537**
C3	.105	.042	.118	**.786**	-.007	.136	.140	**.682**
C4	.066	.154	.175	**.780**	.224	-.049	.263	**.711**
C5	.016	.065	.253	**.666**	.115	.186	-.214	**.675**
C6	**.366**	-.098	.139	**.312**	.051	.099	.048	**.710**
C7	**.317**	.265	**.345**	**.315**	.154	**.484**	-.051	**.553**
D1	**.406**	.222	.239	.032	**.463**	.051	**.567**	-.066
D2	**.779**	.226	.117	.076	**.889**	.127	.191	.210
D3	**.682**	.124	.073	.265	**.873**	.137	.073	.129
D4	**.868**	-.022	.108	-.009	**.842**	-.050	.261	.105
D5	**.491**	**.403**	.240	-.128	-.046	**.416**	**.448**	.037
D6	**.832**	.089	.096	-.029	**.872**	.133	.182	.058
D7	**.838**	-.008	.129	-.076	**.724**	.120	-.081	.197

^*^The percentage of the variance explained by the whole model appears in parentheses.

^†^The percentage of the variance explained by each factor appears in parentheses.

^‡^Bold type identifies .30 or larger loads.

### SCL-90-R

Table 4 shows reliability data for the SCL-90-R. The Cronbach alpha of the global severity index (GSI) was .96 and .97 for the paper-and-pencil and online versions, respectively. Scales showed .72 or higher except for phobic anxiety in the paper-and-pencil questionnaire, which was .62. Test-retest correlation ranged from .63 for hostility to .86 for psychoticism. The correlation for the GSI was .83.

**Table 4:** SCL-90-R reliability results: Cronbach alpha, mean differences t test, Pearson test-retest correlation, and squared eta effect size

	**Paper-and-Pencil**		**Online**				
**SCL-90-R**	**Mean (SD)**	α	**Mean (SD)**	α	***t* (*P* value)**	***r* (*P* value)**	**η^2^**
Somatization	.58 (.55)	.85	.39 (.41)	.82	5.10 (< .001)	.73 (< .001)	.208
Obsessive-Compulsive	.61 (.59)	.84	.45 (.50)	.85	4.10 (< .001)	.74 (< .001)	.145
Interpersonal sensitivity	.62 (.51)	.78	.45 (.51)	.83	5.54 (< .001)	.83 (< .001)	.236
Depression	.66 (60)	.88	.53 (.62)	.92	2.91 (.004)	.74 (< .001)	.079
Anxiety	.47 (.46)	.81	.36 (.38)	.79	3.30 (.001)	.70 (< .001)	.099
Hostility	.40 (.43)	.72	.29 (.41)	.76	3.01 (.003)	.63 (< .001)	.084
Phobic anxiety	.18 (.31)	.62	.15 (.29)	.72	1.56 (.123)	.84 (< .001)	.024
Paranoid ideation	.42 (.51)	.73	.34 (.49)	.81	2.41 (.017)	.77 (< .001)	.056
Psychoticism	.20 (.33)	.75	.19 (.32)	.76	.812 (.419)	.86 (< .001)	.007
GSI (Total)	.50 (.40)	.96	.37 (.36)	.97	5.47 (< .001)	.83 (< .001)	.232

Paper-and-pencil means were higher than online means in every score. A *t* test for repeated measures showed that those differences were statistically significant except for phobic anxiety and psychoticism. Squared eta (η^2^) analysis showed values from small to medium. It is important to note that η^2^ for the GSI was .232, which means that more than 23% of the variance was due to method administration (see Table 4). That proportion could have clinical implications that we will discuss later.

The factorial analysis showed difficulty confirming the expected nine-factors solution for both the online and paper-and-pencil administration. All the items where scattered through the forced nine factors without the presupposed order. As an example, we could mention that the first online factor grouped items (.30 or bigger loads) from seven theoretical scales (anxiety, hostility, depression, interpersonal sensitivity, obsessive-compulsive symptoms, phobic anxiety, and psychoticism). Another fact lead us to reject a factorial analysis for this questionnaire: the KMO test (online = .394; paper-and-pencil = .414) was under the recommended .6 value to accept such an analysis [32]. As a comparison, the GHQ-28 KMO values were .788 for the online version and .781 for the paper-and-pencil version. As a result we do not recommend the use of the SCL-90-R scales as the way to discriminate among different clinical problems.

However, it should be noted that the first unrotated component of the analysis explained more than 25% of the variance in both online and paper-and-pencil questionnaires, and 94% of the online items (85 out 90) and 92% of the paper-and-pencil ones (83 out 90) presented loads of .30 or higher for this general factor. This, together with reliability data, led us to accept this test as a general screening tool.

## Discussion

The aim of this work was to find out whether the psychometric characteristics of two well-known, self-report questionnaires remain consistent when administered via the Internet. Our analysis of the online versions matches the results of the paper-and-pencil versions in several aspects, but some identified differences between the two formats should be explained.

### GHQ-28

Regarding the GHQ-28, internal consistency was high in both formats (Cronbach alpha for all scales and total score was over .70). Nevertheless, test-retest reliability ranged from a too modest .30 to .72, while other studies have presented coefficients over .70, some of them using Spanish translations of the questionnaire [33]. On one hand, it could be said that the GHQ-28 keeps its reliability as internal consistency when delivered via the Internet, but, on the other hand, equivalence data are lower than expected, especially in scale C. The small test-retest correlation in this scale (.30) as well as its factorial instability in the paper-and-pencil version could be due to the experimental situation. C scale accounts for “social dysfunction,” and the paper-and-pencil situation was “social” (all the students and the investigator were together in the same classroom), whereas the online task was completed at home. Perhaps this caused participants to interpret the C items differently and to vary their answers.

Mean differences between formats were small enough to be negligible if we take into account η^2^ results. Even in scale B, where these differences were significant, the accounted variance for method administration was only 5.7%, a proportion not very important when talking about a rough general screening test.

Validity analysis of GHQ-28 showed that previously reported factor structure was fairly replicated. As a whole, both online and paper-and-pencil results of this study match former works in which scales C (social dysfunction) and D (depression) were more consistent than A (somatic symptoms) and B (anxiety) [33,34]. This situation is clinically understandable given that somatic symptoms are frequent in anxiety disorders. A tentative explanation for the relative instability of the online C factor based on the experimental situation has already been pointed out.

### SCL-90-R

The SCL-90-R maintained its internal consistency when delivered over the Internet; in fact, it was higher than in the paper-and-pencil version, and test-retest correlations were as high as in previous studies [26]. This leads us to propose equivalence of the online and paper-and-pencil formats. Our results match the literature on reliability as internal consistency in nonclinical samples [26] as well as the equivalence data using an SCL-90-R computerized version [17]. Nevertheless, all paper-and-pencil scores were higher than online ones. Here it is important to mention the η^2^ values. Three scale differences and that for the GSI could be labeled as medium effects. As we mentioned above, in the case of GSI, this means that 23.2% of the variance could be explained by test administration method. This proportion is big enough to recommend caution if we mixed online and traditional versions of this test because scores could differ enough as to cover (if online is first) or to resemble (if paper is first) the effect of a treatment. The presence of the experimenter and the participants during the paper-and-pencil session, plus the fact that all participants had Internet connections at home, leads us to believe that the online tasks were less aversive. This could be a tentative explanation of higher paper-and-pencil scores.

We have already mentioned the problems that most authors have faced when replicating the nine-factor structure of the SCL-90-R. In our case, the more parsimonious interpretation matches the conclusions of several articles: even when the proposed solution has more than a factor [22], the high variance percentage explained by the first factor should lead to consideration of the total score as a general dimension of psychopathology [26]. Perhaps, as stated by Cyr et al [23], “interpreting nine dimensions for clinical purposes is highly questionable” no matter if we are talking about online or classic assessment. As only one strong factor appears, a psychopathology discrimination function can not be assigned to this tool. However, it does not lose its usefulness as a general psychopathological screening tool.

### Conclusion

The results of this research are encouraging for the online use of the two questionnaires. In the GHQ-28, although two of its four scales had relatively small equivalence values, those of the other two as well as that of the general score were adequate, and the internal consistency values were high. Further research should be carried out to confirm this data, but our work supports the online use of this assessment tool.

The same could be said about the SCL-90-R: its online version could be taken as being equivalent to its classic paper-and-pencil version, and its internal consistency is high. However, paper-and-pencil scores were higher than online ones. Even when an online test has shown acceptable reliability and validity values, the use of normative data from paper-and-pencil questionnaires may not be appropriate [2], suggesting that as online testing spreads, research to obtain a bank of normative data from larger Internet samples should be an important goal.

Factorial analysis results for both online questionnaires showed factor structures similar to paper-and-pencil versions.  SCL-90-R showed a similar factorial structure in its online and paper-and-pencil applications, but the results do not replicate the nine factor structure proposed by Derogatis  [21]. Other researchers also had difficulties to replicate the nine factors  [21,23,26]. As a consequence, we recommend use of the questionnaire as a general index of psychopathology, using the summary score (GSI) only, not the subscales.

The use of standardized tools administered through the Internet needs further investigation, and as for paper-and-pencil versions, they are not enough to properly assess a clinical case. The results obtained by these screening tools should be taken only as part of the assessment and should never be used as the only basis to support any intervention.

Lastly, we should mention two limitations of this work that future research should try to address. First, as the most probable Internet users, the university community will be one of the target populations for any Internet-related research. We must stress that this technology is spreading fast, so samples outside the university community must be analyzed. Second, our experimental design did not allow us to separate the effects of the test-retest situation from those of the format effect. Therefore, the next step should be to compare four groups (Internet and Internet; Internet and paper-and-pencil; paper-and-pencil and paper-and-pencil; paper-and-pencil and Internet) to discriminate both effects.

## Multimedia Appendix

Psychological assessment through the Internet 
		      	

## Abbreviations

GHQ-28: General Health Questionnaire-28
GSI: global severity index
SCL-90-R: Symptoms Check-List-90 Revised

## References

[1] Carlbring P, Nilsson-Ihrfelt E, Waara J, Kollenstam C, Buhrman M, Kaldo V, Söderberg M, Ekselius L, Andersson G (2005). Treatment of panic disorder: live therapy vs. self-help via the Internet.

[2] Buchanan Tom (2003). Internet-based questionnaire assessment: appropriate use in clinical contexts. Cogn Behav Ther.

[3] Carlbring P, Brunt S, Bohman S, Richards P, Öst L-G, Andersson G (2007). Internet vs. paper and pencil administration of questionnaires commonly used in panic/agoraphobia research. Comput Human Behav.

[4] Booth-Kewley S, Larson GE, Miyoshi DK (2007). Social desirability effects on computerized and paper-and-pencil questionnaires. Comput Human Behav.

[5] Suler John (2004). The online disinhibition effect. Cyberpsychol Behav.

[6] Schulenberg SE, Yutrzenka BA (2004). Ethical issues in the use of computerized assessment. Comput Human Behav.

[7] Bressani RV, Downs AC (2002). Youth independent living assessment: testing the equivalence of web and paper/pencil versions of the Ansell-Casey Life Skills Assessment. Comput Human Behav.

[8] Surís A, Borman PD, Lind L, Kashner TM Aggression, impulsivity, and health functioning in a veteran population: equivalency and test-retest reliability of computerised and paper-and-pencil administrations. Comput Human Behav.

[9] Pouwer F, Snoek F J, Van Der Ploeg H M, Heine R J, Brand A N (1998). A comparison of the standard and the computerized versions of the Well-being Questionnaire (WBQ) and the Diabetes Treatment Satisfaction Questionnaire (DTSQ). Qual Life Res.

[10] Ritter Philip, Lorig Kate, Laurent Diana, Matthews Katy (2004). Internet versus mailed questionnaires: a randomized comparison. J Med Internet Res.

[11] Buss A H, Perry M (1992). The aggression questionnaire. J Pers Soc Psychol.

[12] Herrero J, Menese J (2006). Short Web-based versions of the perceived stress (PSS) and Center for Epidemiological Studies-Depression (CESD) scales: a comparison to pencil and paper responses among Internet users. Comput Human Behav.

[13] Epstein J, Klinkenberg WD, Wiley D, McKinley L (2001). Insuring sample equivalence across Internet and paper-and-pencil assessments. Comput Human Behav.

[14] Goldberg D P, Hillier V F (1979). A scaled version of the General Health Questionnaire. Psychol Med.

[16] Vallejo MA, Jordán CM, Mañanes G, Vasallo A, Comeche MI Clinical psychology offers in the Internet in Spain. Comput Human Behav.

[17] Schmitz N, Kruse J, Heckrath C, Alberti L, Tress W (1999). Diagnosing mental disorders in primary care: the General Health Questionnaire (GHQ) and the Symptom Check List (SCL-90-R) as screening instruments. Soc Psychiatry Psychiatr Epidemiol.

[18] Aderibigbe Y A, Riley W, Lewin T, Gureje O (1996). Factor structure of the 28-item general health questionnaire in a sample of antenatal women. Int J Psychiatry Med.

[19] Iwata N, Saito K (1992). The factor structure of the 28-item General Health Questionnaire when used in Japanese early adolescents and adult employees: age- and cross-cultural comparisons. Eur Arch Psychiatry Clin Neurosci.

[20] Kilic C, Rezaki M, Rezaki B, Kaplan I, Ozgen G, Sagduyu A (1997). Ozturk MO General Health Questionnaire (GHQ-12 & GHQ-28): psychometric properties and factor structure of the scales in a Turkish primary care sample. Soc Psychiatry Psychiatr Epidemiol.

[21] Derogatis LR, Cleary PA (1977). Confirmation of the dimensional structure of the SCL-90: a study in construct validation. J Clin Psychol.

[22] Brophy C J, Norvell N K, Kiluk D J (1988). An examination of the factor structure and convergent and discriminant validity of the SCL-90R in an outpatient clinic population. J Pers Assess.

[23] Cyr J J, McKenna-Foley J M, Peacock E (1985). Factor structure of the SCL-90-R: is there one?. J Pers Assess.

[24] Martínez-Azumendi O, Fernández-Gómez C, Beitia-Fernández M (2001). Factorial variance of the SCL-90-R in a Spanish out-patient psychiatric sample. Actas Esp Psiquiatr.

[25] Rauter U K, Leonard C E, Swett C P (1996). SCL-90-R factor structure in an acute, involuntary, adult psychiatric inpatient sample. J Clin Psychol.

[26] Schmitz N, Hartkamp N, Kiuse J, Franke G H, Reister G, Tress W (2000). The Symptom Check-List-90-R (SCL-90-R): a German validation study. Qual Life Res.

[27] INE (Instituto Nacional de Estadística) Estadística de la enseñanza universitaria en España.

[28] Lobo A, Muñoz PE (1996). Cuestionario de salud general. GHQ (General Health Questionnaire). Guía para el usuario de las distintas versiones. Versiones en lengua española validadas.

[29] González de Rivera JL, Derogatis LR, De Las Cuevas C, Garcia-Marco R, Rodríguez F, Henry R, Monterrey AL (1989). The Spanish version of the SCL-90-R. Normative data in the general population.

[30] Vacha-Haase T, Thompson B (2004). How to estimate and interpret various effect sizes. J Couns Psychol.

[31] Cohen J (1968). Multiple regression as a general data-analytic system. Psychol Bull.

[32] Pardo A, Ruiz MA (2002). SPSS 11.Guía para el análisis de datos.

[33] Gibbons PF, Hilda M (2004). Assessment of the factor structure and reliability of the 28-item version of the General Health Questionnaire (GHQ-28) in El Salvador. Int J Clin Health Psychol.

[34] Werneke U, Goldberg D P, Yalcin I, Ustün B T (2000). The stability of the factor structure of the General Health Questionnaire. Psychol Med.

