# Supplementary material for: Psychological Assessment via the Internet: A Reliability and Validity Study of Online (vs Paper-and-Pencil) Versions of the General Health Questionnaire-28 (GHQ-28) and the Symptoms Check-List-90-Revised (SCL-90-R)
Source: J Med Internet Res. 2007 Jan 31;9(1):e2. doi: 10.2196/jmir.9.1.e2 (PMC1794673; doi:10.2196/jmir.9.1.e2)
Supplement: Supplementary file 1 [file jmir_v9i1e2_app1.ppt]

## Slide 1
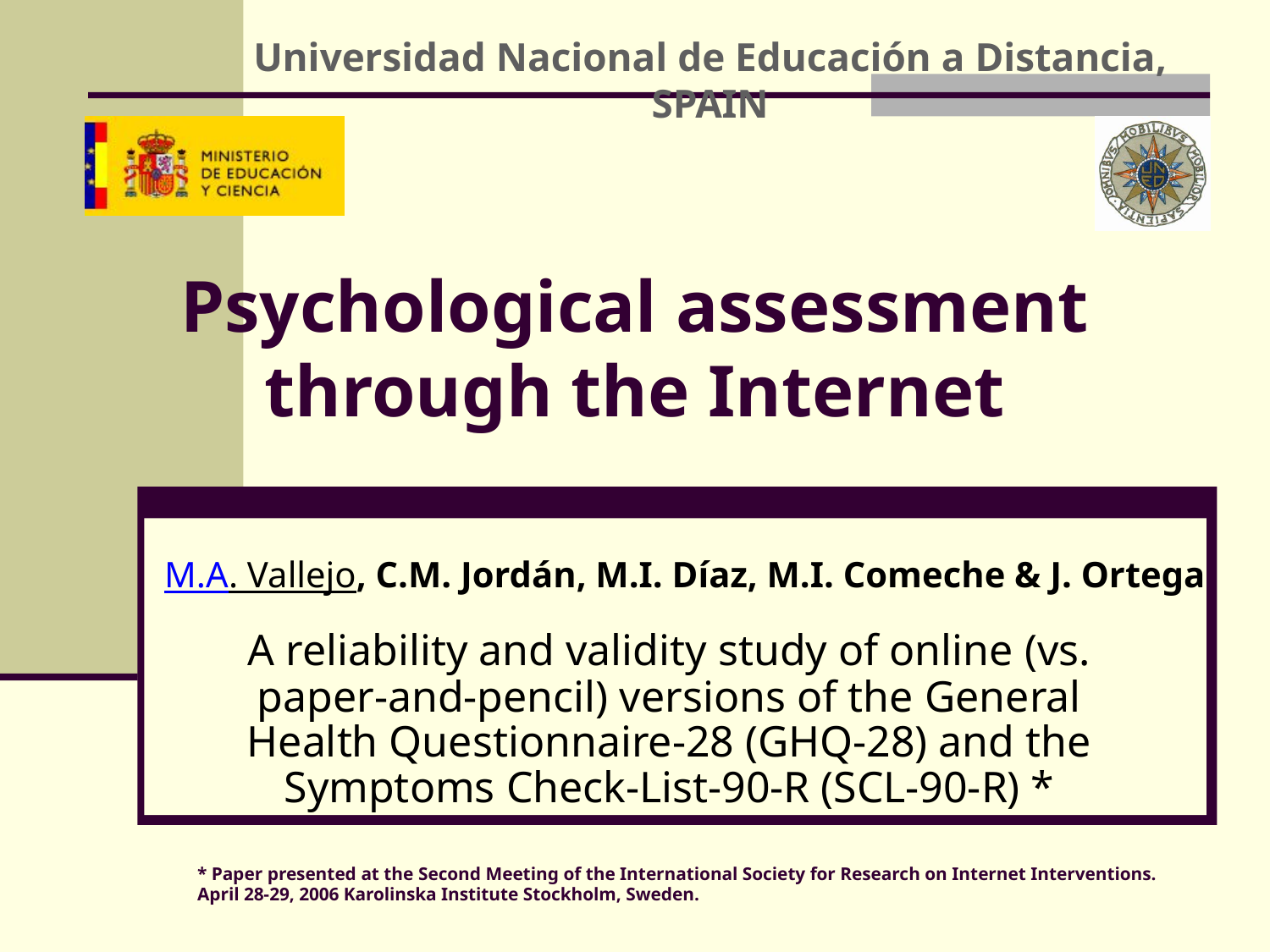

Universidad Nacional de Educación a Distancia, SPAIN
# Psychological assessment through the Internet
M.A. Vallejo, C.M. Jordán, M.I. Díaz, M.I. Comeche & J. Ortega
A reliability and validity study of online (vs. paper-and-pencil) versions of the General Health Questionnaire-28 (GHQ-28) and the Symptoms Check-List-90-R (SCL-90-R) *
* Paper presented at the Second Meeting of the International Society for Research on Internet Interventions. April 28-29, 2006 Karolinska Institute Stockholm, Sweden.

## Slide 2
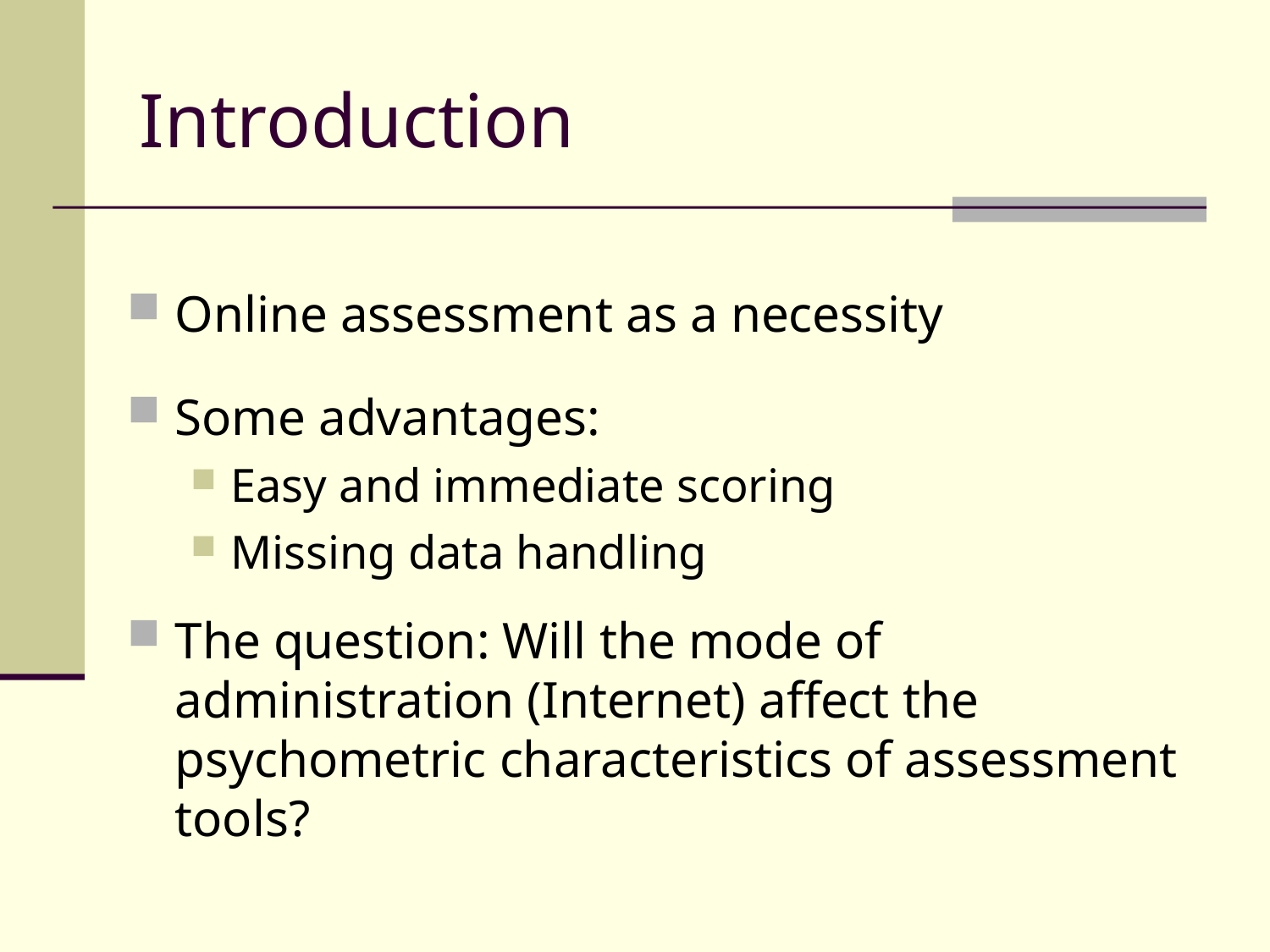

# Introduction
Online assessment as a necessity
Some advantages:
Easy and immediate scoring
Missing data handling
The question: Will the mode of administration (Internet) affect the psychometric characteristics of assessment tools?

## Slide 3
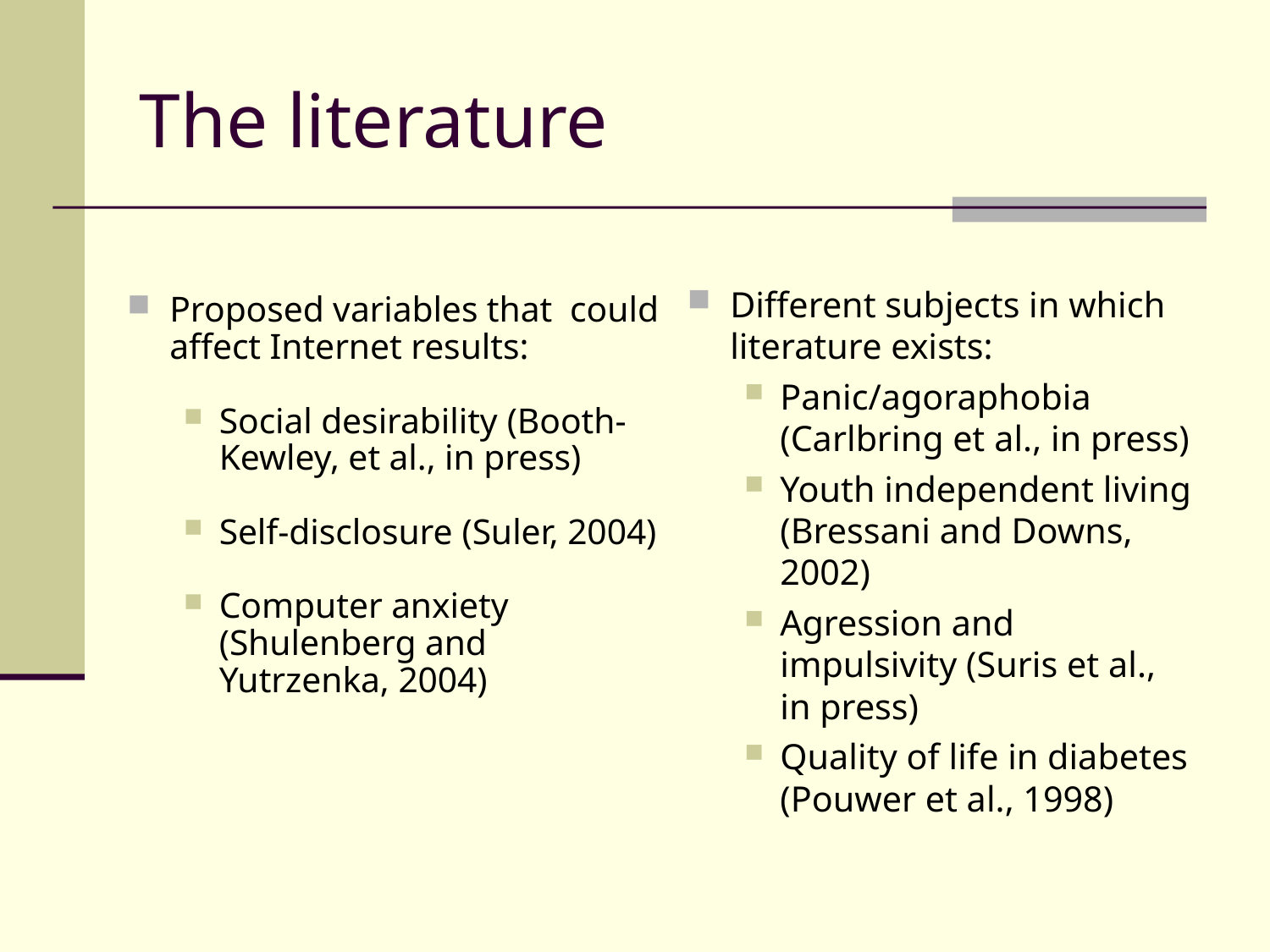

# The literature
Different subjects in which literature exists:
Panic/agoraphobia (Carlbring et al., in press)
Youth independent living (Bressani and Downs, 2002)
Agression and impulsivity (Suris et al., in press)
Quality of life in diabetes (Pouwer et al., 1998)
Proposed variables that could affect Internet results:
Social desirability (Booth-Kewley, et al., in press)
Self-disclosure (Suler, 2004)
Computer anxiety (Shulenberg and Yutrzenka, 2004)

## Slide 4
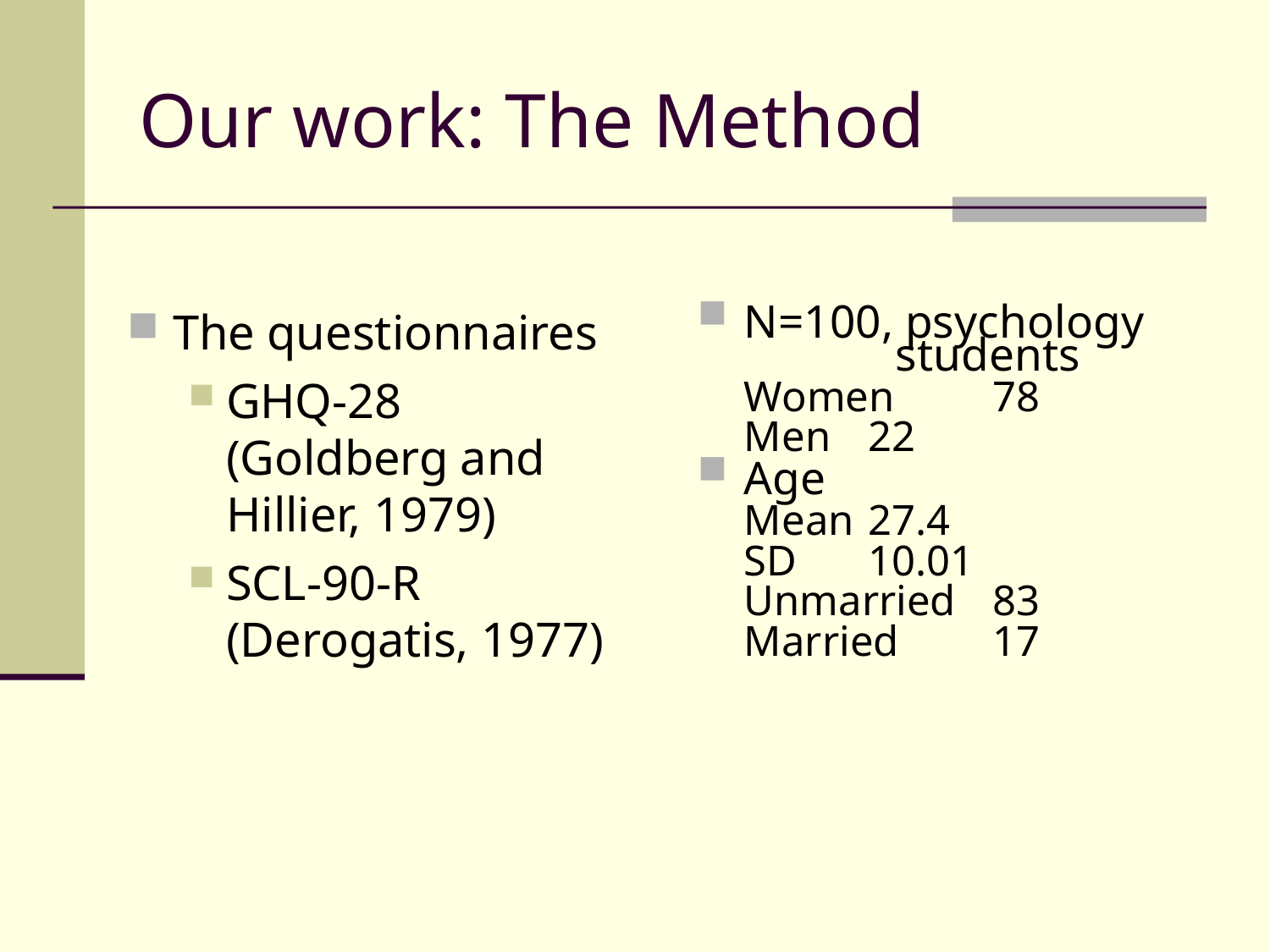

# Our work: The Method
The questionnaires
GHQ-28 (Goldberg and Hillier, 1979)
SCL-90-R (Derogatis, 1977)
N=100, psychology
 students
		Women	78
		Men		22
Age
		Mean		27.4
		SD		10.01
		Unmarried	83
		Married	17

## Slide 5
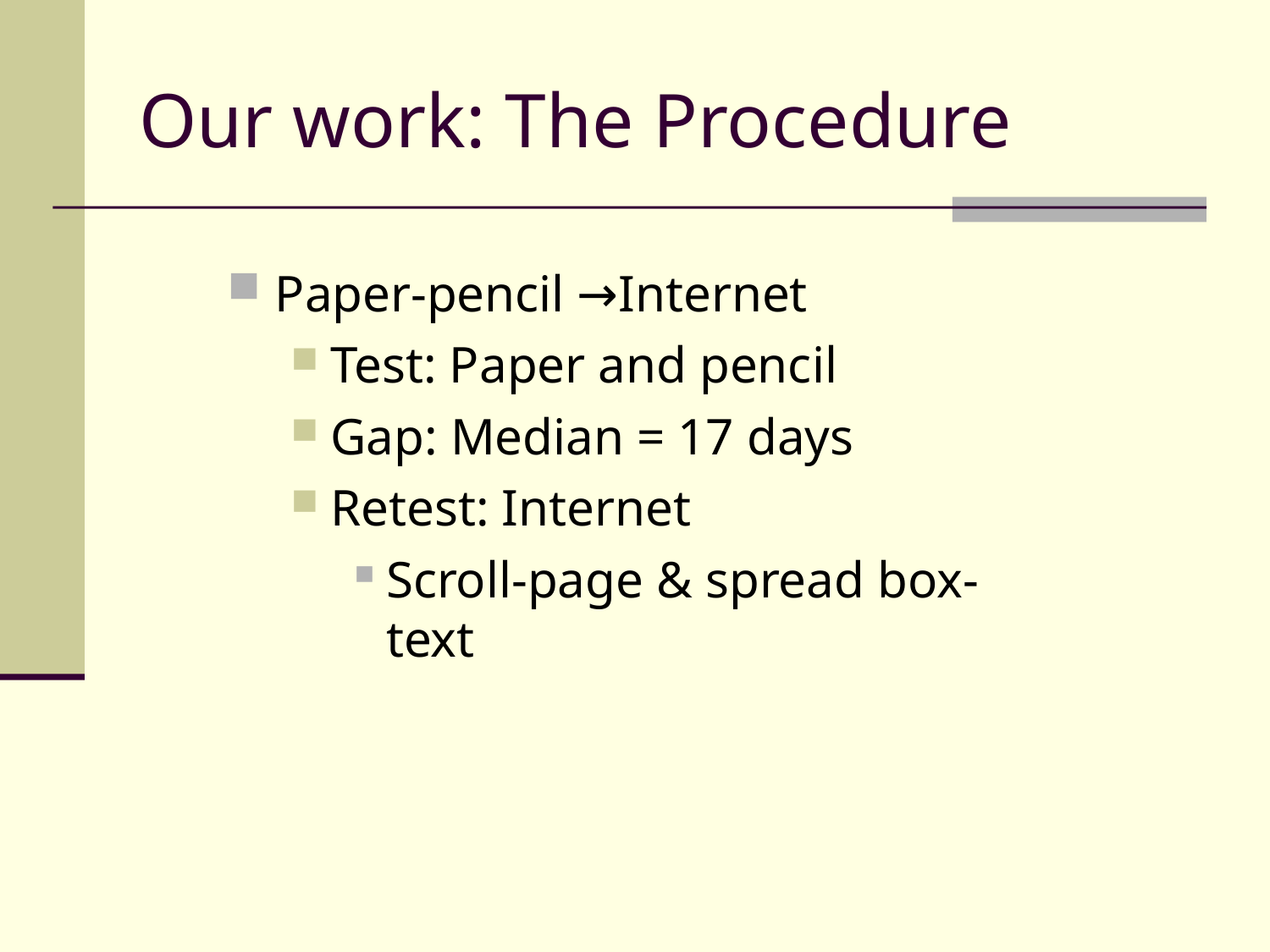

# Our work: The Procedure
Paper-pencil →Internet
Test: Paper and pencil
Gap: Median = 17 days
Retest: Internet
Scroll-page & spread box-text

## Slide 6
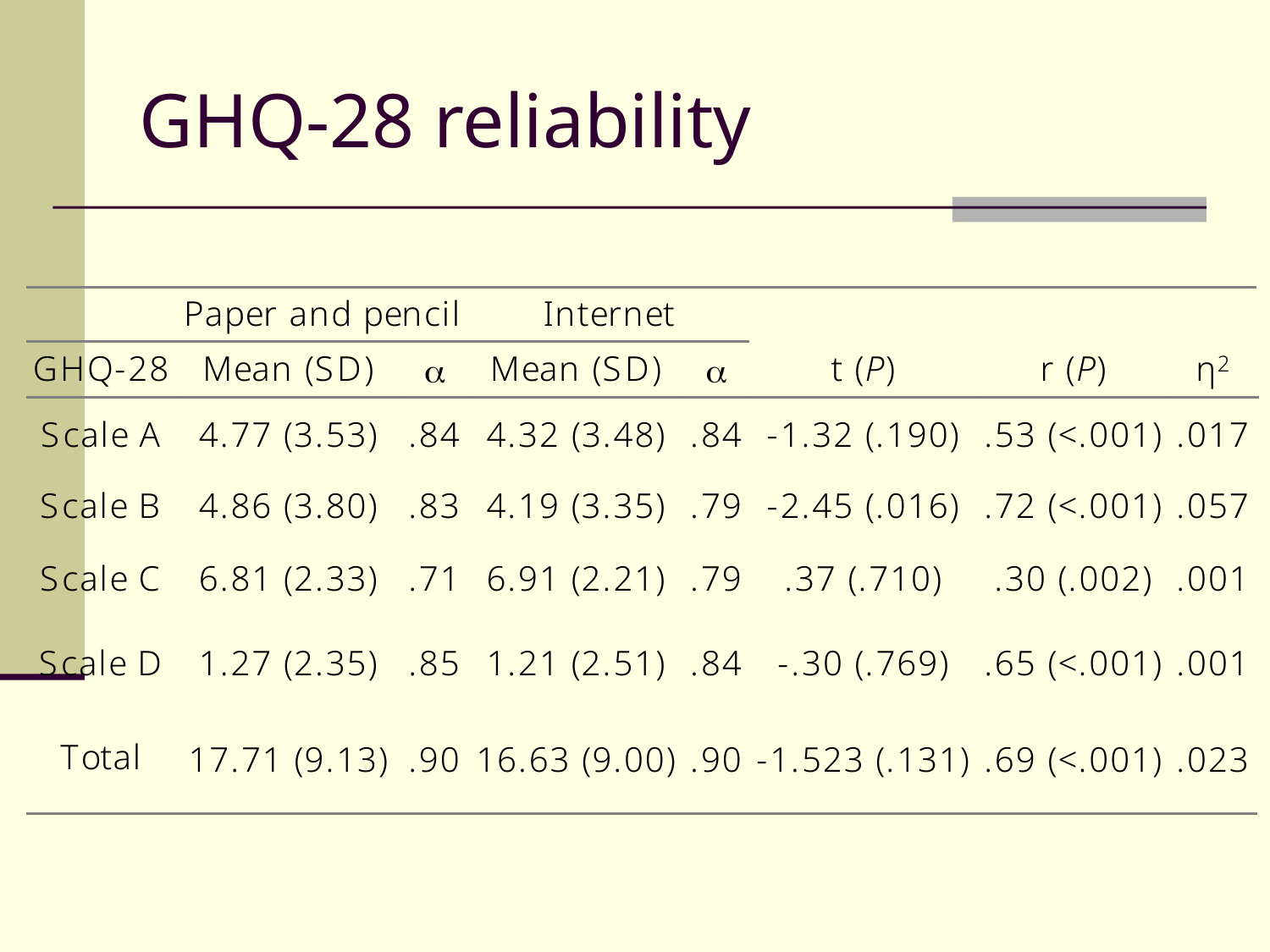

# GHQ-28 reliability

## Slide 7
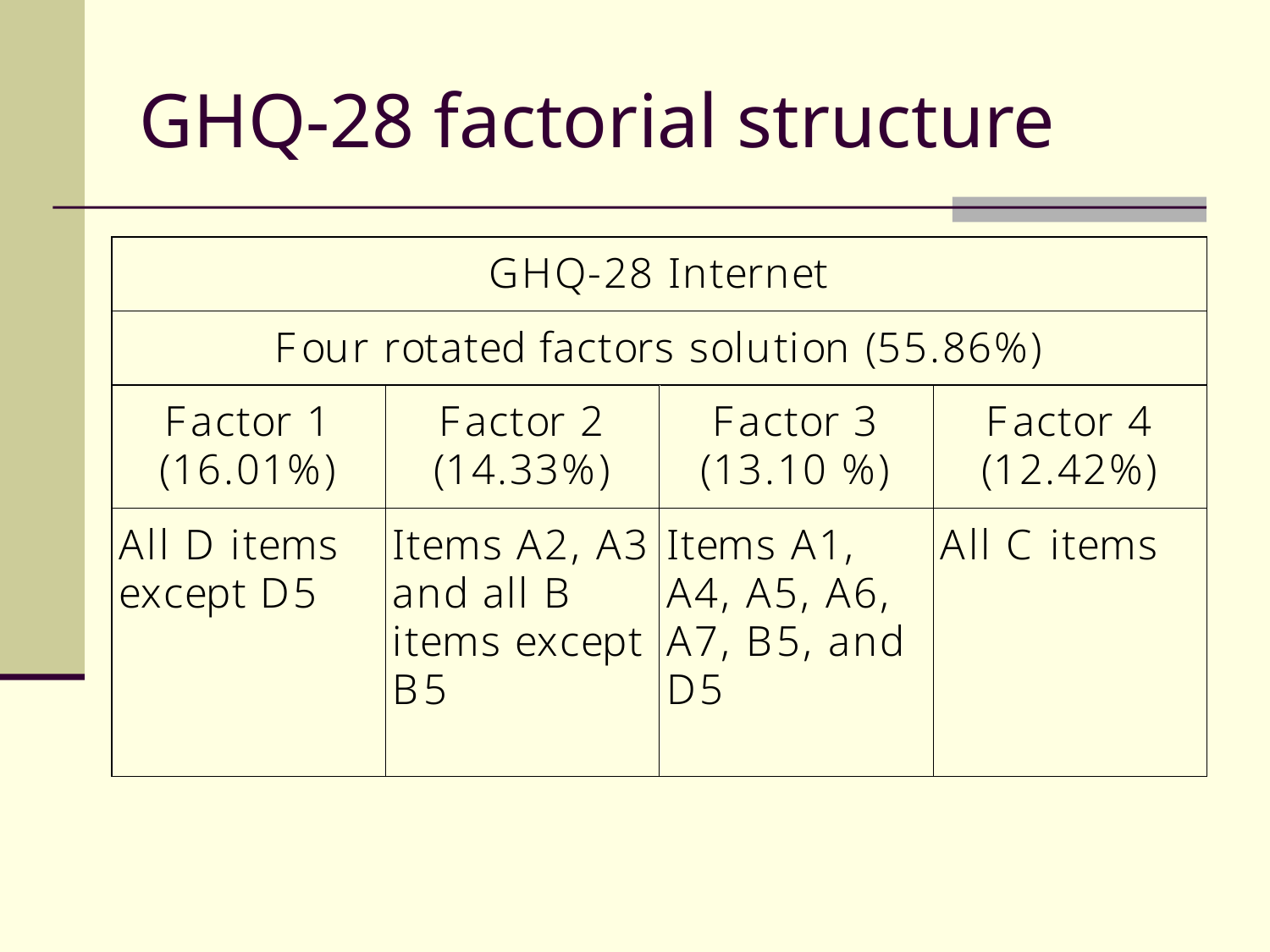

# GHQ-28 factorial structure

## Slide 8
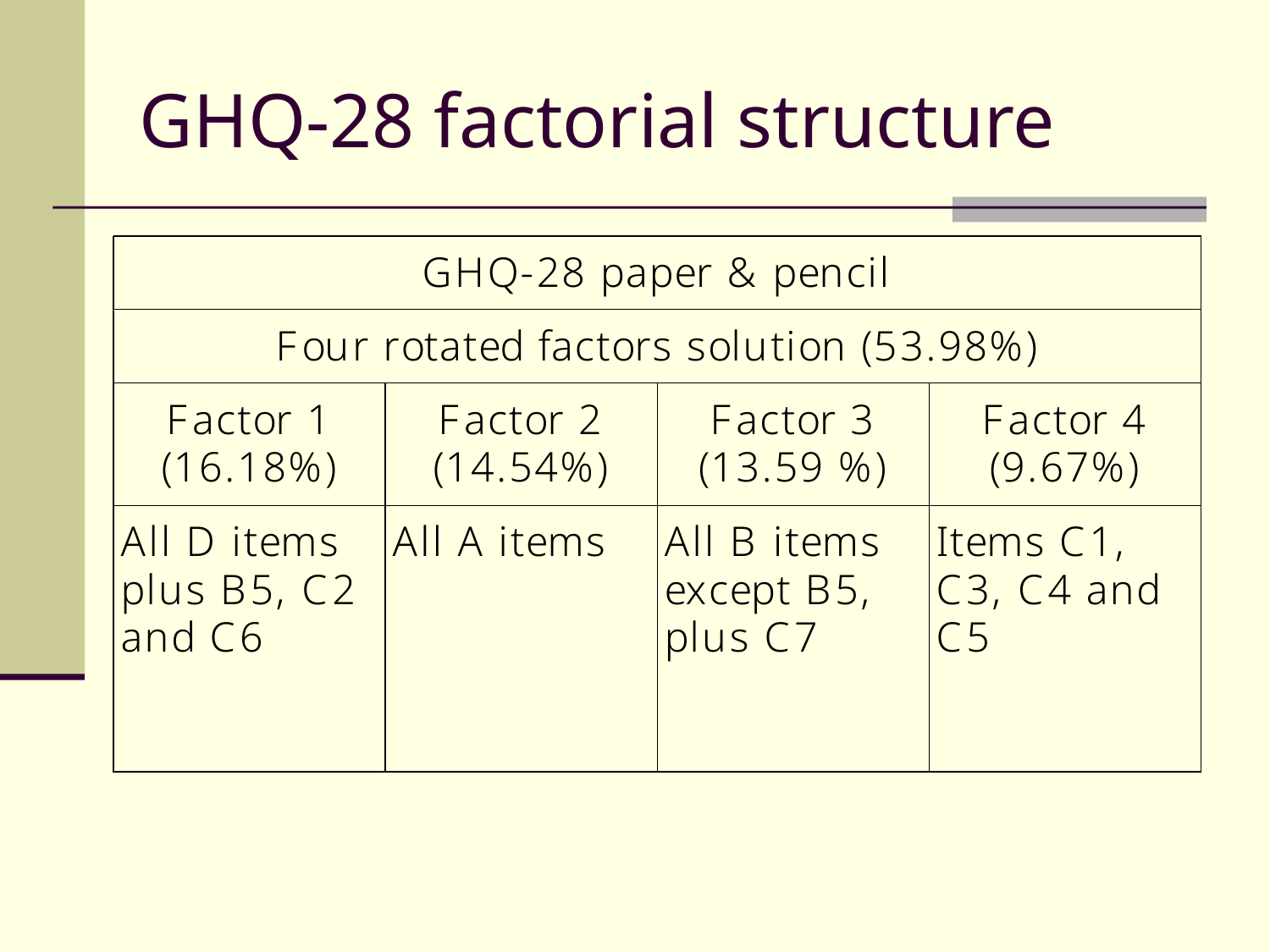

# GHQ-28 factorial structure

## Slide 9
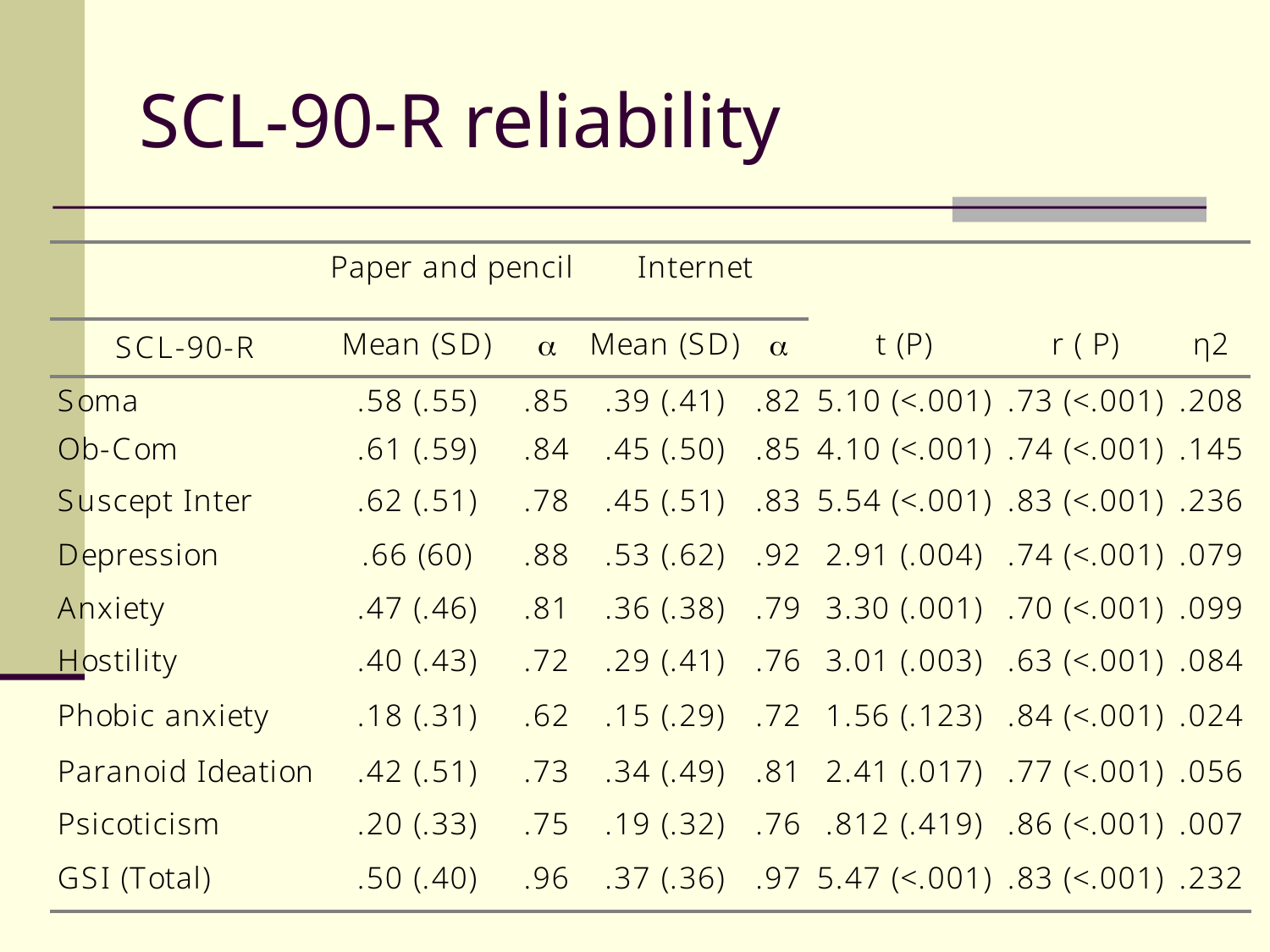

# SCL-90-R reliability

## Slide 10
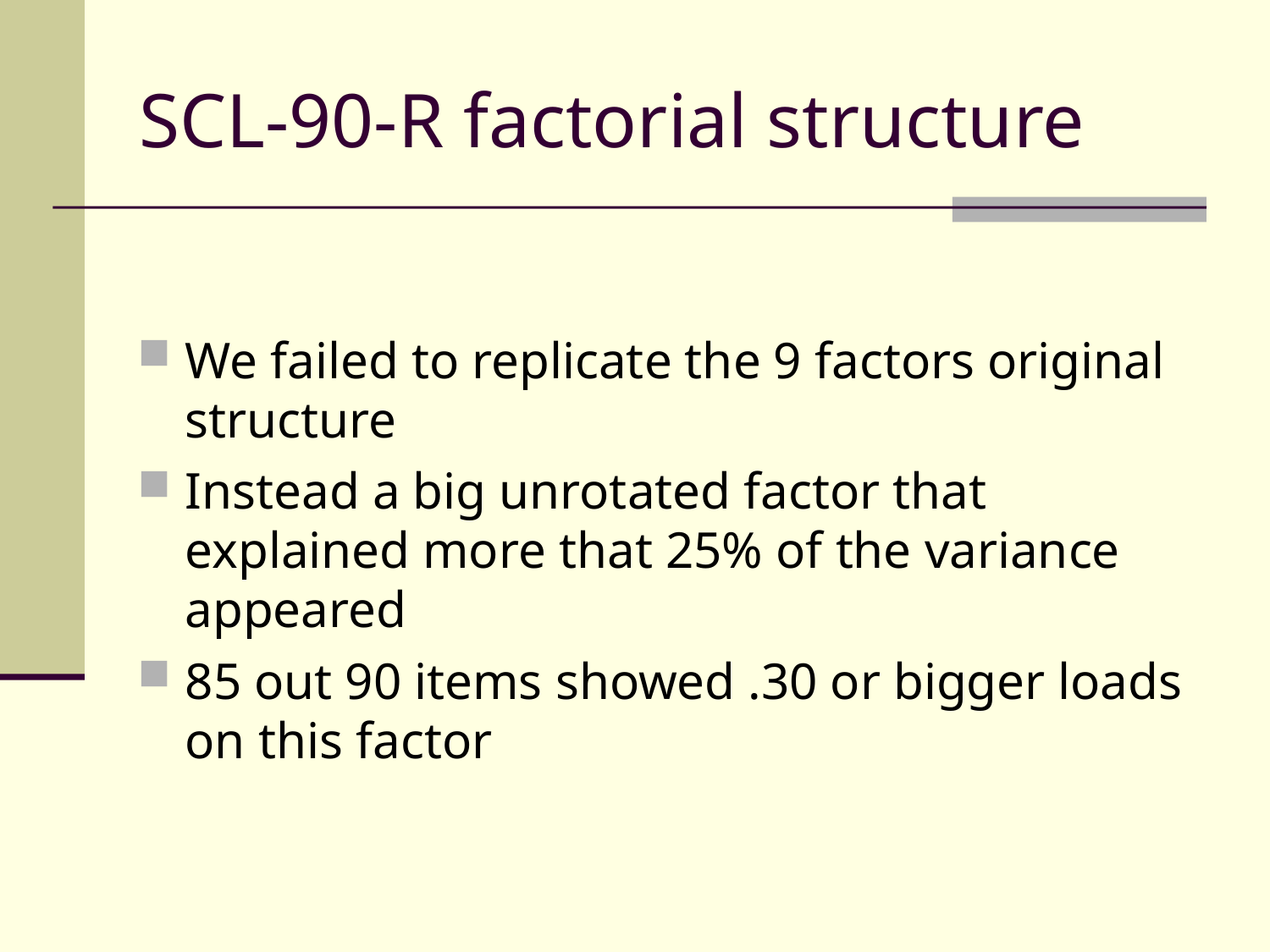

# SCL-90-R factorial structure
We failed to replicate the 9 factors original structure
Instead a big unrotated factor that explained more that 25% of the variance appeared
85 out 90 items showed .30 or bigger loads on this factor

## Slide 11
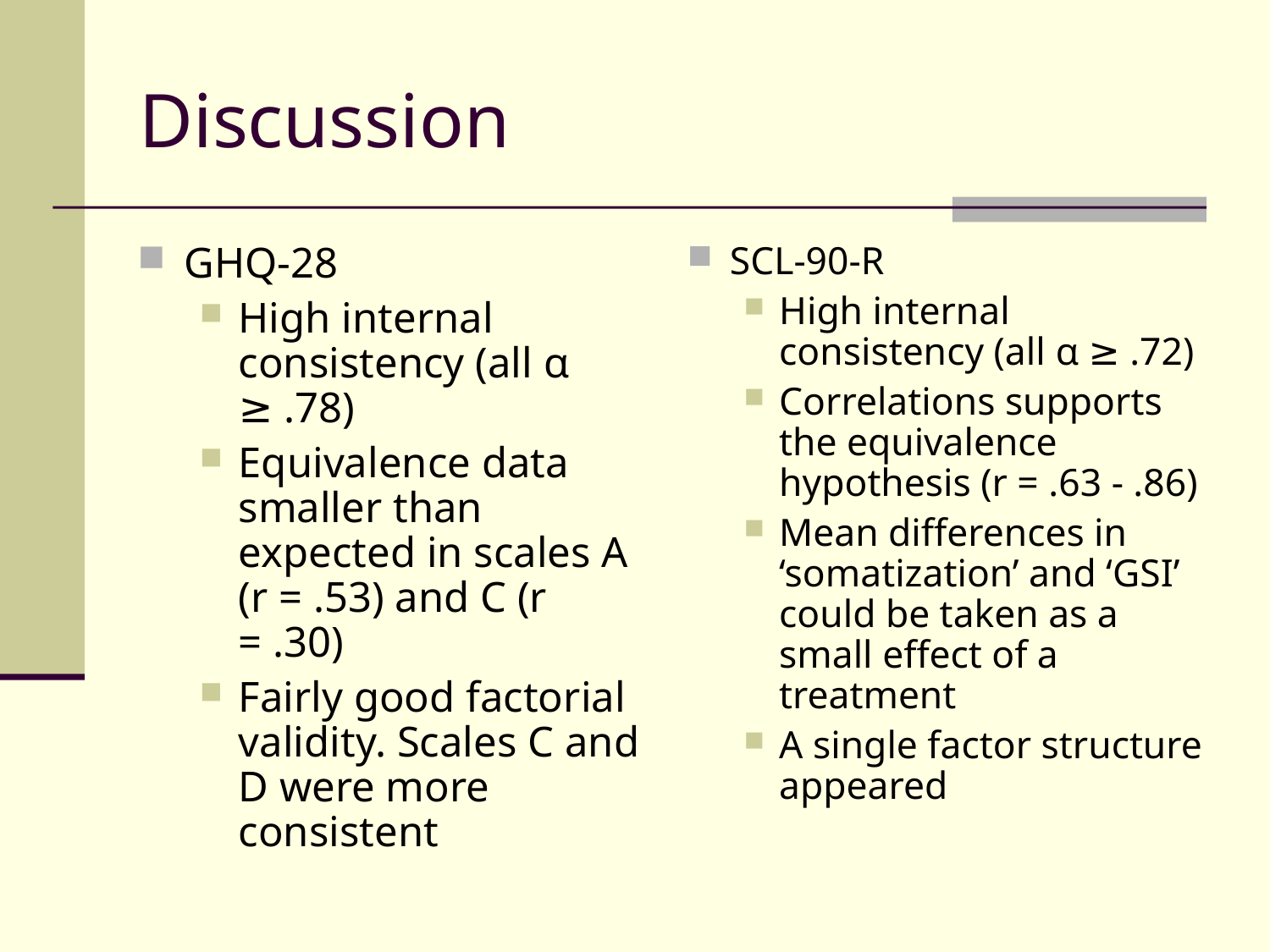

# Discussion
GHQ-28
High internal consistency (all α ≥ .78)
Equivalence data smaller than expected in scales A (r = .53) and C (r = .30)
Fairly good factorial validity. Scales C and D were more consistent
SCL-90-R
High internal consistency (all α ≥ .72)
Correlations supports the equivalence hypothesis (r = .63 - .86)
Mean differences in ‘somatization’ and ‘GSI’ could be taken as a small effect of a treatment
A single factor structure appeared

## Slide 12
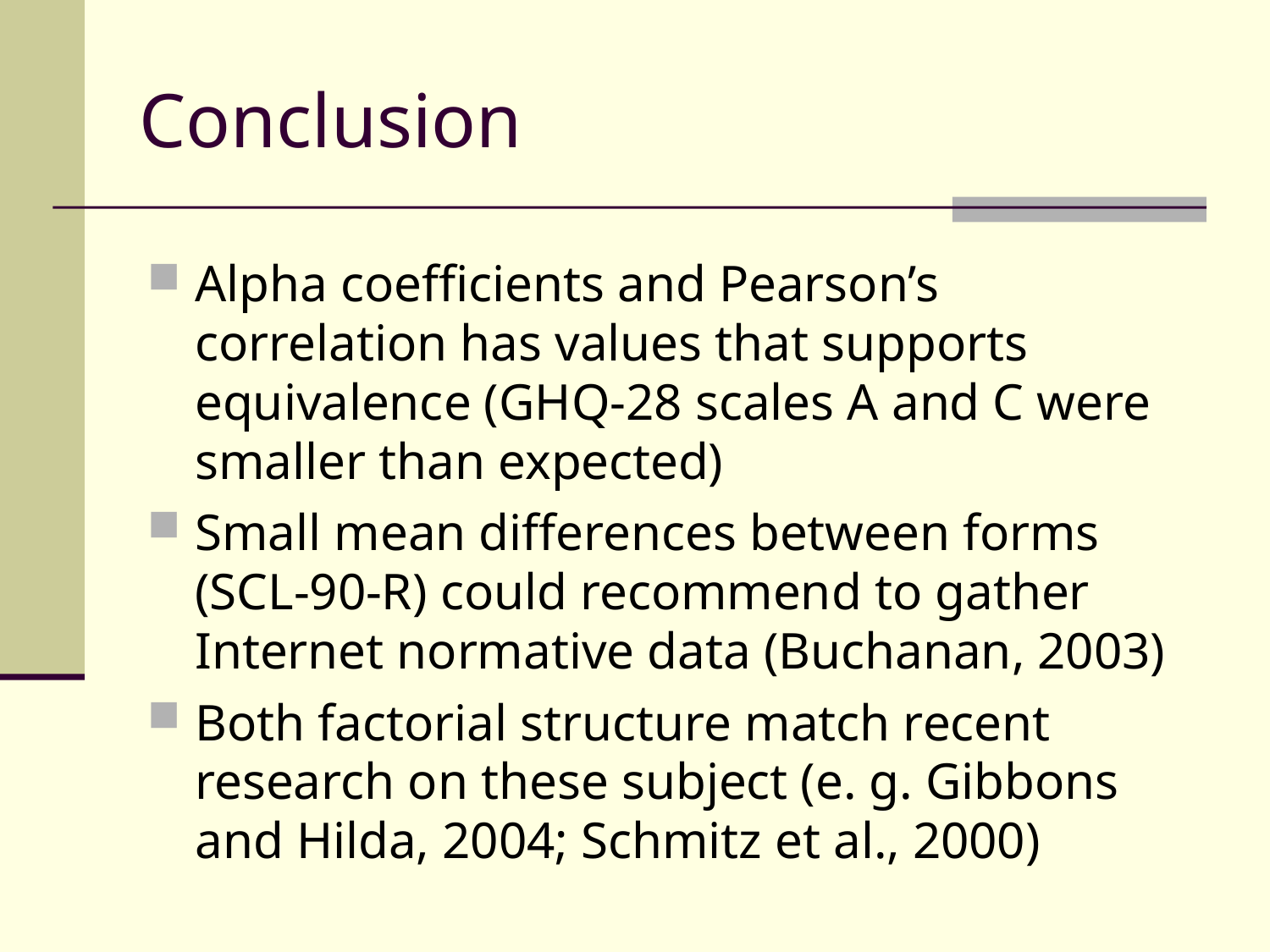

# Conclusion
Alpha coefficients and Pearson’s correlation has values that supports equivalence (GHQ-28 scales A and C were smaller than expected)
Small mean differences between forms (SCL-90-R) could recommend to gather Internet normative data (Buchanan, 2003)
Both factorial structure match recent research on these subject (e. g. Gibbons and Hilda, 2004; Schmitz et al., 2000)

## Slide 13
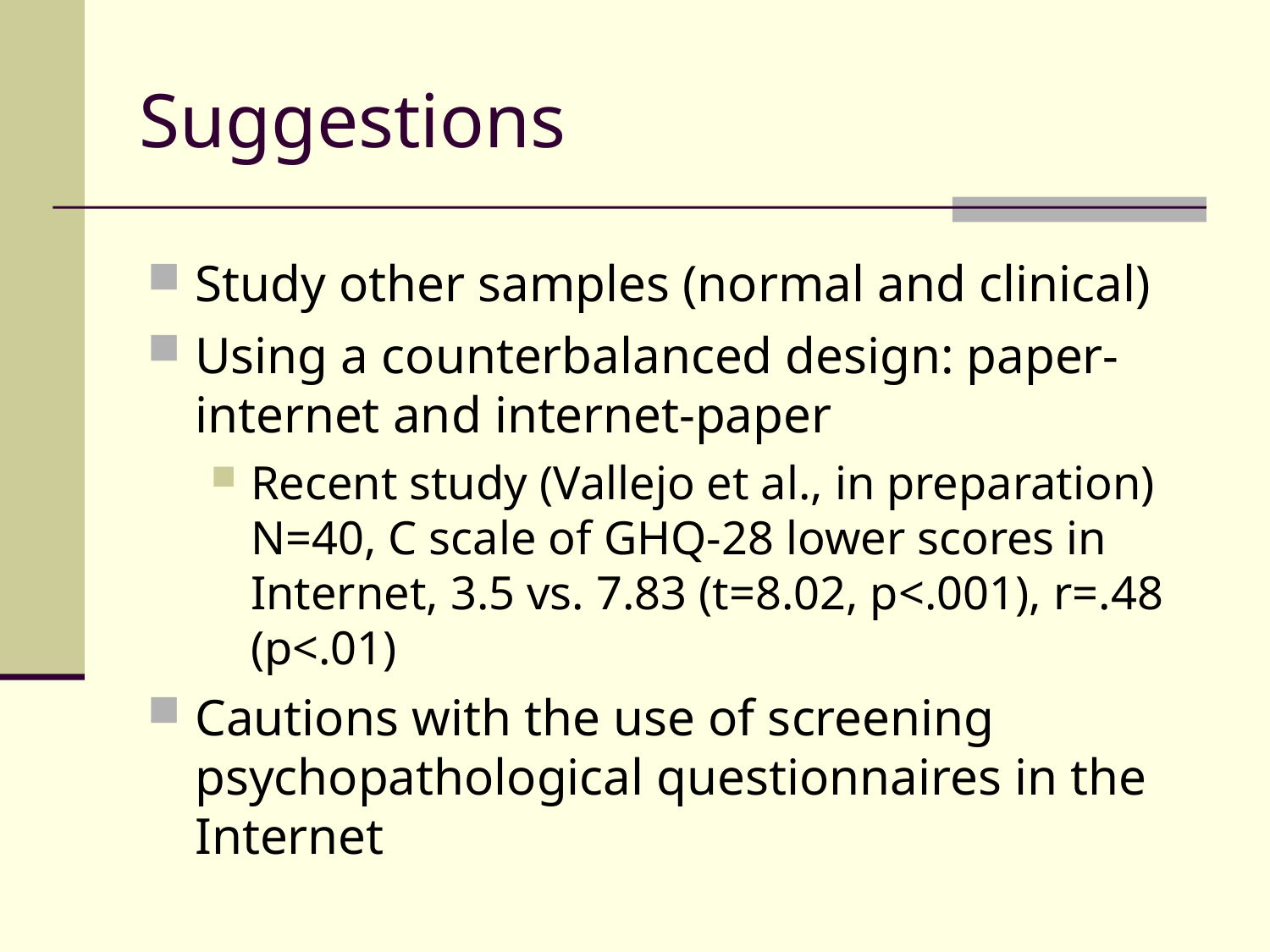

# Suggestions
Study other samples (normal and clinical)
Using a counterbalanced design: paper-internet and internet-paper
Recent study (Vallejo et al., in preparation) N=40, C scale of GHQ-28 lower scores in Internet, 3.5 vs. 7.83 (t=8.02, p<.001), r=.48 (p<.01)
Cautions with the use of screening psychopathological questionnaires in the Internet

## Slide 14
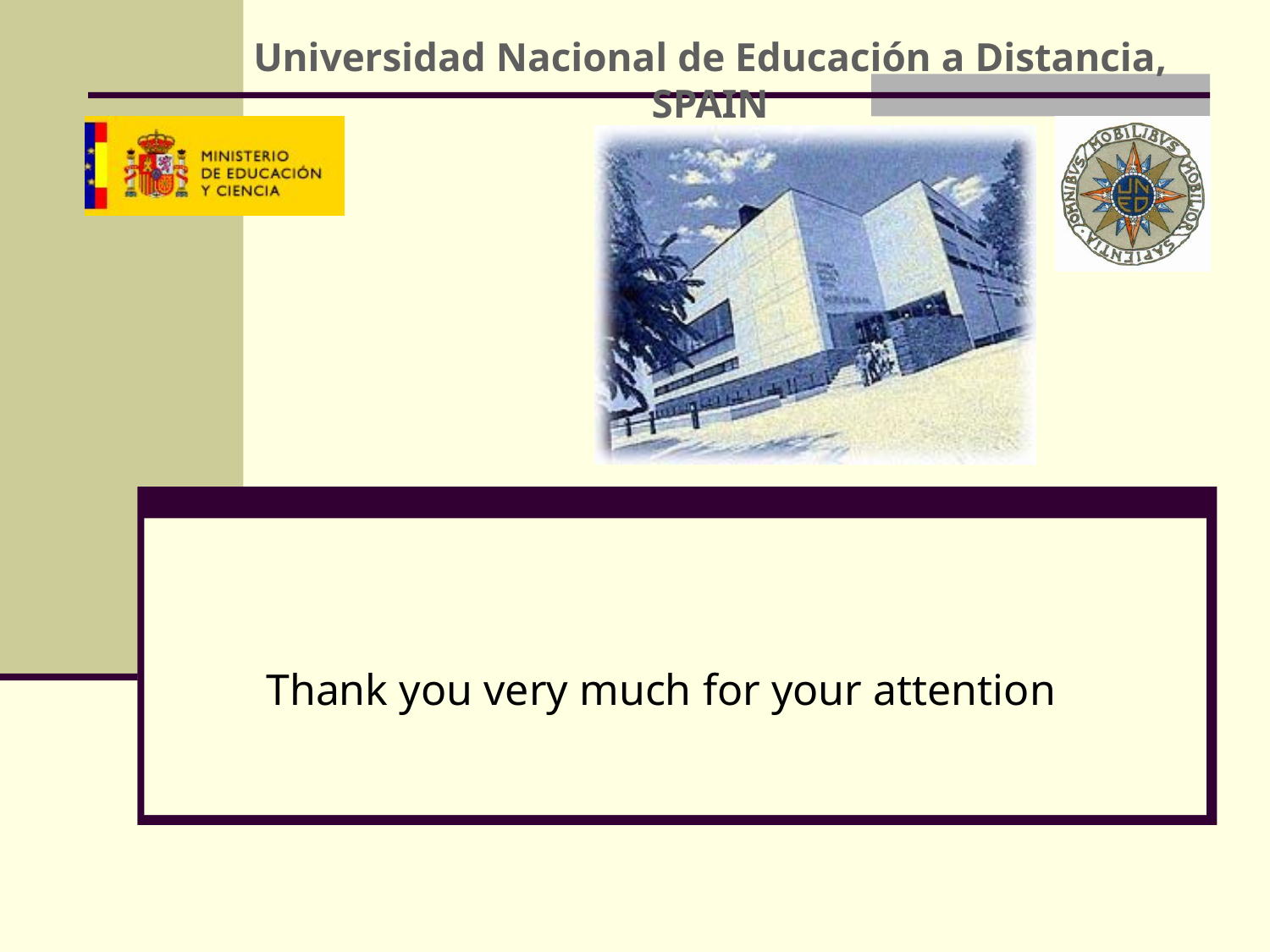

Universidad Nacional de Educación a Distancia, SPAIN
# Thank you very much for your attention
